# Antibody-Dependent Respiratory Burst against *Plasmodium falciparum* Merozoites in Individuals Living in an Area with Declining Malaria Transmission

**DOI:** 10.3390/vaccines12020203

**Published:** 2024-02-16

**Authors:** Doreen D. Mutemi, James Tuju, Rodney Ogwang, Lydia Nyamako, Kennedy M. Wambui, Ivette R. Cruz, Pär Villner, Victor Yman, Samson M. Kinyanjui, Ingegerd Rooth, Billy Ngasala, Anna Färnert, Faith H. A. Osier

**Affiliations:** 1 Division of Infectious Diseases, Department of Medicine Solna, Karolinska Institutet, 171 77 Stockholm, Sweden; 2 Department of Parasitology and Medical Entomology, Muhimbili University of Health and Allied Sciences, Dar es Salaam 11102, Tanzania; 3 Centre for Geographic Medicine Research (Coast), Kenya Medical Research Institute–Wellcome Trust Research Programme, Kilifi 80108, Kenya; 4 Epidemiology and Biostatistics Division, School of Public Health, University of the Witwatersrand, Johannesburg 2000, South Africa; 5 Division of Biostatistics, Karolinska Institutet, 171 77 Stockholm, Sweden; 6 Department of Infectious Diseases, Södersjukhuset, 118 61 Stockholm, Sweden; 7 Pwani University Bioscience Research Centre, Pwani University, Kilifi 80108, Kenya; 8 Centre for Tropical Medicine and Global Health, Nuffield Department of Medicine, University of Oxford, Oxford OX3 7LG, UK; 9 School of Business Studies, Strathmore University, Nairobi 0200, Kenya; 10 Nyamisati Malaria Research Group, Pwani 61621, Tanzania; 11 Department of Women’s and Children’s Health, International Maternal and Child Health, Uppsala University, 751 05 Uppsala, Sweden; 12 Department of Infectious Diseases, Karolinska University Hospital, 171 76 Stockholm, Sweden; 13 Centre of Infectious Diseases, Heidelberg University Hospital, 69120 Heidelberg, Germany; 14 Department of Life Sciences, Imperial College London, London SW7 2AZ, UK

**Keywords:** *Plasmodium falciparum*, malaria, transmission, ADRB, immunity, vaccines

## Abstract

Malaria transmission intensity affects the development of naturally acquired immunity to malaria. An absolute correlate measure of protection against malaria is lacking. However, antibody-mediated functions against *Plasmodium falciparum* correlate with protection against malaria. In children, antibody-mediated functions against *P. falciparum* decline with reduced exposure. It is unclear whether adults maintain antibody-mediated functions as malaria transmission declines. This study assessed antibody-dependent respiratory burst (ADRB) in individuals from an area with declining malaria transmission. In an age-matched analysis, we compare ADRB activity during high versus low malaria transmission periods. Age significantly predicted higher ADRB activity in the high (*p* < 0.001) and low (*p* < 0.001) malaria transmission periods. ADRB activity was higher during the high compared to the low malaria transmission period in older children and adults. Only older adults during the high malaria transmission period had their median ADRB activity above the ADRB cut-off. Ongoing *P. falciparum* infection influenced ADRB activity during the low (*p* = 0.01) but not the high (*p* = 0.29) malaria transmission period. These findings propose that naturally acquired immunity to *P. falciparum* is affected in children and adults as malaria transmission declines, implying that vaccines will be necessary to induce and maintain protection against malaria.

## 1. Introduction

*Plasmodium falciparum* is responsible for more than 90% of all estimated malaria cases and deaths globally [1]. With the emergence of resistance to artemisinin, developing effective malaria vaccines against *P. falciparum* has become increasingly important. The RTS, S/AS01 (Mosquirix™), the first-ever malaria vaccine based on *P. falciparum* circumsporozoite protein (CSP), suffers low efficacy [2] with waning anti-CSP antibodies post-vaccination [3]. The R21/Matrix-M™, another malaria vaccine based on the same sporozoite antigen [4], was recently recommended by the WHO for malaria prevention in children [5]. In a randomised, phase 3 trial among children aged 5–36 months followed for 24 months, R21/Matrix-M™ prevented 75% of malaria episodes, and there was a 77% reduction in multiple malaria episodes in the vaccinated group compared to the control group in children from sites with seasonal malaria transmission [6]. Unless pre-erythrocytic stage malaria vaccines confer sterile immunity, developing effective blood-stage malaria vaccines and identifying blood-stage antigen vaccine candidates to complement pre-erythrocytic stage malaria vaccines in multi-antigen/multi-stage malaria vaccines [7] is crucial to eliminating the burden of malaria.

Understanding naturally acquired immunity to malaria could pave the way to developing efficacious malaria vaccines. Successfully treating malaria-infected children using passively transferred immunoglobulin G (IgG) from adult residents in malaria-endemic settings suggests that antibodies are critical mediators of naturally acquired immunity to malaria [8,9]. To date, no unanimously accepted antibody-mediated correlate measure of protection against malaria exists. Studies have mainly focused on antibody levels and breadth against *P. falciparum* antigens [10]. The ability of antibodies to neutralize *P. falciparum* in vitro in growth inhibition assays has been widely assessed as a correlate of protection against malaria. However, inconsistent results have been reported [11]. Antibody-mediated functions against *P. falciparum* based on their ability to recruit cells of the innate immune system by their Fc-portion have recently gained attention.

Neutrophils are the most abundant white blood cells in the human peripheral blood and are regulated as the first line of defense of the innate immune system [12,13]. Opsonizing antibodies against *P. falciparum* can recruit neutrophils by their Fc-portion, releasing reactive oxygen species (ROS) that destroy the parasite [14]. This antibody-mediated function can be measured in an in vitro solid-phase chemiluminescence assay called the antibody-dependent respiratory burst (ADRB), where isoluminol is used to detect extracellular ROS released by neutrophils [15,16]. ADRB has been correlated with protection against clinical malaria [17].

Naturally acquired immunity to malaria is influenced by malaria transmission intensity; in areas with high malaria transmission, young children are mainly susceptible to severe malaria, while older children and adults are not [18]. Thus, immunity to severe malaria appears to be acquired relatively quickly during the first years of life. In contrast, children and adults are at risk of clinical malaria episodes in low malaria transmission areas, and immunity appears to be acquired more slowly [18].

Over the past two decades, global malaria transmission trends have fluctuated. WHO estimated a decrease of 13 million malaria cases globally in 2014 compared to 2000. Global malaria cases increased in 2015, with the most significant increase of 11 million estimated cases between 2019 and 2020. In 2022, there was an estimated increase of 5 million cases compared to 2021 [1]. In children, malaria antibodies and antibody-mediated functional activity generally decrease with the reduction in malaria transmission [19,20,21]. Even in the context of RTS, S vaccination, antibody magnitude, and antibody-mediated functional activity significantly reduce post-vaccination in children [22,23]. There is limited knowledge of the influence of changing malaria transmission on antibody magnitude in adults. The few studies conducted among adults mainly span one to three malaria transmission periods [24,25]. Whether functional antibody activity is maintained in adults in the context of declining malaria transmission has not been studied. To better understand naturally acquired immunity to malaria, we aim to assess ADRB in an area of declining malaria transmission over 25 years in children and adults. To our current knowledge, it is the first time that data on antibody-mediated functional activity against *P. falciparum* has been reported in the context of declining malaria transmission in the same area over an extended period in children and adults. Other studies have assessed antibody-mediated functions by comparing data from two villages with differing malaria transmission intensities [16,26], and another study assessed antibody-mediated functions against *P. falciparum* in children at three time points over a year as malaria transmission decreased [20].

## 2. Materials and Methods

### 2.1. Study Area and Population

Nyamisati is a rural village located in the mangrove swamps of the Rufiji River Delta in the Kibiti District of Tanzania. Several cross-sectional surveys to assess the epidemiology of malaria have been conducted in Nyamisati from 1986 to 2019, where demographic characteristics, axillary body temperature, venous blood, and blood smears were collected. Reports from these studies revealed a gradual decline from hyperendemic to hypoendemic malaria transmission intensity [27]. The decrease in malaria transmission may be attributed to several factors, including a research team closely monitoring malaria from 1985 to 1991 and 1993 to 1999, where diagnosis and treatment were readily available. The team then moved but repeated cross-sectional surveys were ongoing from 1993 to 2019, where the research team distributed bed nets during the 1999, 2010, 2016, and 2019 cross-sectional surveys. Other factors that may have contributed to the decline in malaria transmission in Nyamisati include the introduction of rapid malaria diagnosis kits, bed net distribution campaigns, and the use of artemisinin-based combination therapy (ACT) as a first-line malaria treatment.

The present study is based on six cross-sectional surveys where venous blood was collected into EDTA tubes from which plasma and packed cells were separated and frozen at −20 °C and later stored at −80 °C for long-term storage. The samples are from the 1993, 1994, and 1995 cross-sectional surveys, referred to as the high malaria transmission period, and from the 2016, 2018, and 2019 cross-sectional surveys, referred to as the low malaria transmission period. Individuals were selected from the high and low malaria transmission periods matched by age.

### 2.2. Parasite Detection by Microscopy

Blood from a pricked finger or EDTA tube was placed on a labeled glass slide to prepare a thin and thick smear. The slides were air-dried, and the thin smear was fixed by dipping it in absolute methanol for a few seconds. They were then stained with 10% Giemsa for 20 min. The slides were then rinsed with a gentle flow of buffered water until the stain was removed. They were then dried and read using a light microscope at 100× magnification using oil immersion. The slide was declared negative when no parasites were observed over 100 fields. Parasites were counted per 200 white blood cells, assuming 8000 white blood cells per microliter of blood, to estimate their density.

### 2.3. Parasite Detection by Real-Time PCR

Real-time PCR was used to detect four Plasmodium species simultaneously, i.e., *P. falciparum*, *P. malariae*, *P. ovale* spp., and *P. vivax*. A single reaction was performed using species-specific probes, forward primers, and conserved reverse primers. The final volume of the reaction mixture was 25 μL, including 0.5 μL DNA, 0.2 μL (10 μmol/L) of each species-specific probe, 0.5 μL *P. falciparum* forward primer, 0.125 μL forward primer of each of the other three species, 0.5 μL (10 μmol/L) of the conserved reverse primer, 0.125 μL TaqMan master mix, Mustang Purple or ROX reference dye, and nuclease-free water. To define the positive samples, a cut-off of 45 cycles was used. The thermal profile started at 95 °C for 20 s, 95 °C for 1 s, and 60 °C for 20 s. Each plate included standards, negative, and species-specific positive controls.

### 2.4. Plasmodium falciparum Parasite Preparation

*P. falciparum* parasites of the 3D7 strain were cultured with 2% hematocrit in O+ human erythrocytes in culture media (RPMI 1640 media supplemented with 30 mM HEPES, 0.05 mg/mL hypoxanthine, 0.025 mg/mL gentamicin, 2 mg/mL D-glucose, 3% *w*/*v* Albumax II, and 7.5% *w*/*v* sodium bicarbonate) and allowed to reach 10–15% parasitemia. Using a magnetic column (Miltenyi Biotec, Bergisch Gladbach, Germany), mature trophozoites were isolated and put back into a culture to enable their development to the schizont stage. A protease inhibitor, Epoxysuccinyl-L-leucylamido (4-guanidino) butane (E64; Sigma-Aldrich, Burlington, MA, USA), was added to allow schizont development to mature schizonts for 6–8 h without rupture. After centrifugation at 1800 rpm for 5 min, the pellet was resuspended with 1 mL of 1×PBS. The number of parasitophorous vacuolar membrane-enclosed merozoite structures (PEMS) was counted using a counting chamber at 1:20 dilution in 1×PBS. PEMS concentration was estimated using the following formula:

PEMS counted × 20 (dilution factor) × 10^4^ (chamber volume) × 1000 = PEMS/mL. (1)

### 2.5. Neutrophils Preparation

Neutrophils were isolated as previously described [28]. Fresh whole blood from healthy Kenyan volunteers was collected in heparin vacutainer tubes. The blood was mixed 1:1 with Hank’s buffered salt solution (HBSS) minus phenol red, Ca^2+^, and Mg^2+^ ions (Sigma-Aldrich, MA, USA) and layered 1:1 over Histopaque 1077 (Sigma-Aldrich, MA, USA) in falcon tubes. The tubes were centrifuged at 600× *g*, 20 °C with no brakes. Following centrifugation, the peripheral blood mononuclear cells (PBMC) and histopaque layer were removed without disturbing the RBC pellet. The RBC pellet was resuspended with 5 mL HBSS and mixed with 3% dextran (Fisher Bioreagent, Chelmsford, MA, USA) by inverting the falcon tube ten times and then incubated at room temperature in the dark for one hour. The supernatant was then carefully collected without disturbing the RBC pellet. The supernatant was then centrifuged for seven minutes at 500× *g* at 4 °C. After centrifugation, the supernatant was discarded. The remaining pellet was treated with 10 mL of ice-cold 0.2% NaCl for 30 s and stopped with 10 mL of ice-cold 1.6% NaCl to lyse the residual RBC contaminant. The falcon tube was centrifuged for 7 min at 500× *g* at 4 °C, and the supernatant was discarded. RBC contaminant was lysed several times to achieve a clear pellet. Once a clear pellet was achieved, it was resuspended with 0.5 mL polymorphonuclear (PMN) buffer (HBSS with 0.1% *w*/*v* BSA (Jackson ImmunoResearch, Baltimore, USA), 1% *w*/*v* D-(+)—Glucose Hybri Max (Sigma-Aldrich, MA, USA), sterile filtered through 0.22 um syringe filter (Pall Life Sciences, New York, USA). The resuspended neutrophils in the PMN buffer were kept on ice until used.

To ensure a neutrophil yield greater than 90%, 5 µL of the resulting suspension was smeared on a slide, fixed with methanol for 10 s, stained with 5% *v*/*v* Giemsa for 10 min, and observed under ×400 magnification, ensuring contamination with RBCs, and debris was less than 10%.

To assess neutrophil viability, 5 µL of the suspension was mixed with 45 µL trypan blue (Sigma-Aldrich, MA, USA) solution, and 15 µL of this was added to a hemocytometer and observed at ×400 under an inverted microscope. Viable cells were identified as not permeable to trypan blue and were at 99–100%. The following formula was used to estimate neutrophil concentration:

Conc. of neutrophils/mL = (number cells in 16 squares) × (dilution factor) × 10^4^ = cells/mL. (2)

The concentration of the PMN was then adjusted accordingly using PMN buffer, ensuring each well was coated with 5.0 × 10^5^ neutrophils.

### 2.6. ADRB Assay

The assay was adapted from Llewellyn et al., 2015 [15]. Briefly, on each well of a Nunc^TM^ MaxiSorp^TM^ 96-well opaque plate, 100 µL of 10 × 10^5^ PEMS was added and incubated at room temperature overnight. Wells were washed three times with 200 µL of 1× PBS per well and then blocked with 200 µL Casein (Thermo Scientific, Waltham, MA, USA) in 1× PBS per well for 1 h at room temperature. Following a wash step, 50 µL plasma samples and controls diluted 1:50 in 1× PBS were added to the plate and incubated at 37 °C for 1 h. Following another washing step, 50 µL of 5 × 10^5^ neutrophils was rapidly added to each well onto the plate, followed by 50 µL of isoluminol at 0.04 mg/mL in 1× PBS. The plate was immediately loaded onto the Biotek synergy plate reader to be read using the Gen 5 software (version 3.00.19) measuring maximum relative light unit (RLU) in each well for 1000 milliseconds every two minutes for 1 h. On each plate, a pool of hyper-immune Kenyan adult sera was used as a positive control and to assign indexed RLU to each sample tested. Six plasma samples from malaria-naïve individuals were used as negative controls.

### 2.7. Data Analysis

Statistical analysis was performed using R and RStudio 2023.06.0+421 (R core team, version 4.1.3 (2022-03-10) [29] and GraphPad Prism (version 9.5.1), where a *p*-value < 0.05 was considered statistically significant for all tests.

Age was grouped into ten categories: 0–4, 5–8, 9–12, 13–16, 17–20, 21–25, 26–30, 31–35, 36–40 and >40 years. Fever was defined as axillary body temperature ≥37.5 °C or febrile in the past 48 h. Symptomatic malaria was defined as fever and *P. falciparum*-positive at sample collection. In contrast, asymptomatic infection was described as an absence of fever but *P. falciparum*-positive at sample collection. We defined the ADRB cut-off as mean plus three standard deviations indexed RLU of six negative controls. The levels of ADRB activity were grouped into three levels of responders by tertiles: high, medium, and low. The Mann–Whitney U test was used to compare differences between medians, and Spearman’s rank correlation was used to assess correlations between ADRB activity and age.

A logistic regression model stratified by malaria transmission was fitted to investigate the association between ADRB activity and parasitemia, adjusting for age (as a continuous variable), sex, and fever.

### 2.8. Ethical Statement

Ethical approval was granted for using the archived plasma samples from cross-sectional surveys in Nyamisati, Tanzania, and for collecting neutrophils from Kenyan volunteers. The institutions that reviewed and granted approval are listed below under the institutional review board statement.

## 3. Results

### 3.1. Demographic Characteristics

Plasma samples from six cross-sectional surveys in Nyamisati, three surveys during the high and three during the low malaria transmission periods, were selected for the study. The characteristics of the study participants from each malaria transmission period are presented in Table 1. There were 329 study participants in total, 158 and 171 from the high and low malaria transmission period, respectively, none of which participated in more than one survey. A similar age distribution, with 16 years as the median age, was observed in both the high and low malaria transmission periods, as shown in Table 1.

### 3.2. Malaria Transmission

In both the high and low malaria transmission periods, real-time PCR was a more sensitive method of detecting Plasmodium parasites than microscopy. No participant was *P. vivax*-positive by real-time PCR, and *P. falciparum* was the dominant species in both malaria transmission periods. *P. falciparum* prevalence by real-time PCR is presented in Table 1.

Symptomatic malaria was limited to children under 12 years during the high malaria transmission period, while asymptomatic infections occurred in all age groups. During the low malaria transmission period, both symptomatic malaria and asymptomatic infections were reported in children and adults, as shown in Supplementary Figure S1.

During the high malaria transmission period, there were more individuals with asymptomatic infections than symptomatic malaria. On the contrary, there was no difference in the proportion of individuals with symptomatic malaria and asymptomatic infections during the low malaria transmission period, as can be seen in Table 1.

In both the high and low malaria transmission period, parasite density was higher among individuals with symptomatic malaria than those with asymptomatic infection. However, only one asymptomatic individual had detectable parasites by microscopy during the low malaria transmission period, as shown in Table 1.

### 3.3. Age and Malaria Transmission Intensity Influence ADRB Activity

**Figure 1:**
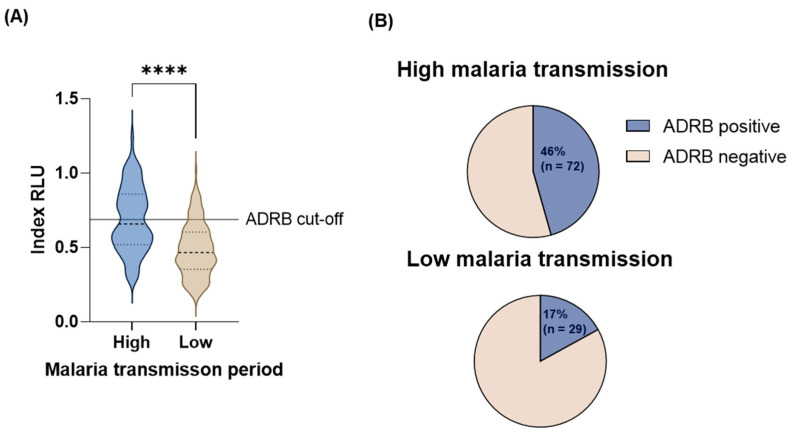
ADRB activity during the high and low malaria transmission period (total high n = 158 and n = 171 in low) (**A**). The magnitude of ADRB as indexed relative light unit (RLU). Medians between groups are compared using the Mann–Whitney U test. ADRB cut-off is indicated by the solid horizontal line and defined as mean plus three standard deviations indexed RLU of six negative controls. On the violins, the Median Index RLU is represented by the thick dotted line, while the 25th and the 75th percentiles are represented by the thin dotted lines. The **** represents *p* < 0.0001 (**B**). The proportion of ADRB-positive and -negative individuals. ADRB positive is defined as individuals with an indexed RLU above the ADRB cut-off, and ADRB-negative is defined as individuals with an indexed RLU equal to and below the ADRB cut-off.

**Figure 2:**
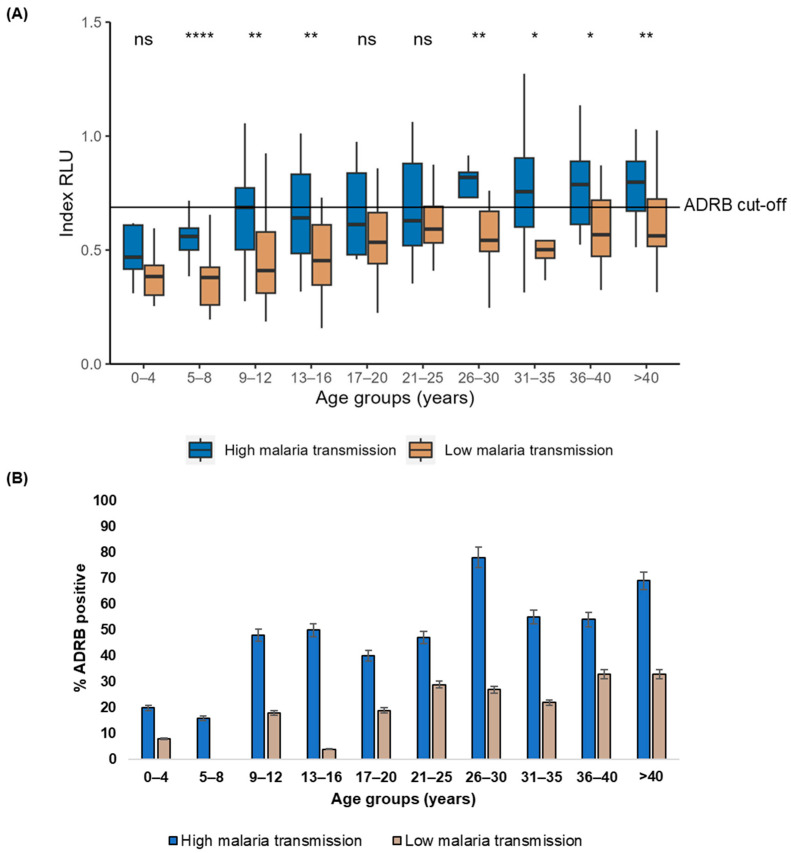
ADRB activity by age categories during the high and low malaria transmission period (**A**). The magnitude of ADRB activity as indexed relative light unit (RLU). The medians between groups are compared using the Mann–Whitney U test. The number (n) of individuals for each age group during the high and low malaria transmission is presented in Table 1. ADRB cut-off is indicated by the solid horizontal line and defined as mean plus three standard deviations indexed RLU of six negative controls. On the boxplots, the Median Index RLU is represented by the thick line, while the 25th and the 75th percentiles are represented by the lines at the lower and upper ends of the box, respectively. The * represents *p* ≤ 0.05, ** represents *p* ≤ 0.01, **** represents *p* < 0.0001, and ns represents *p* > 0.05. (**B**). ADRB proportion positive. Percent (%) ADRB positive is the percentage of individuals with an indexed RLU above the ADRB cut-off. The number (n) of individuals for each age group is presented in Supplementary Table S1.

Overall, higher ADRB activity was observed in the high (median: 0.64 RLU, IQR: 0.51 RLU–0.84 RLU) than in the low (median: 0.48 RLU, IQR: 0.36 RLU–0.62 RLU) malaria transmission period (Mann–Whitney U test, *p* < 0.0001) (Figure 1A). A similar pattern was observed within the age categories; however, ADRB activity was significantly higher in the high versus the low malaria transmission period only in children 5–8 years (*p* < 0.0001), 9–12 years (*p* = 0.001), 13–16 years (*p* = 0.006) and in adults 26–30 years (*p* = 0.006), 31–35 years (0.02), 36–40 years (0.02), and > 40 years (*p* = 0.003) (Figure 2A). A more significant proportion of individuals were ADRB-positive during the high (46%, 95% CI: 38–53%) than in the low (17%, 95% CI: 12–23%) malaria transmission period (Figure 1B); this was also consistent within age categories (Figure 2B).

A positive correlation was observed between ADRB activity and age in both the high (r = 0.44, 95% CI 0.31–0.56, *p* < 0.0001) and low (r = 0.35, 95% CI 0.19–0.48, *p* < 0.0001) malaria transmission periods.

### 3.4. Age, Parasitemia, and Symptomatic Malaria Are Associated with ADRB Activity

**Figure 3:**
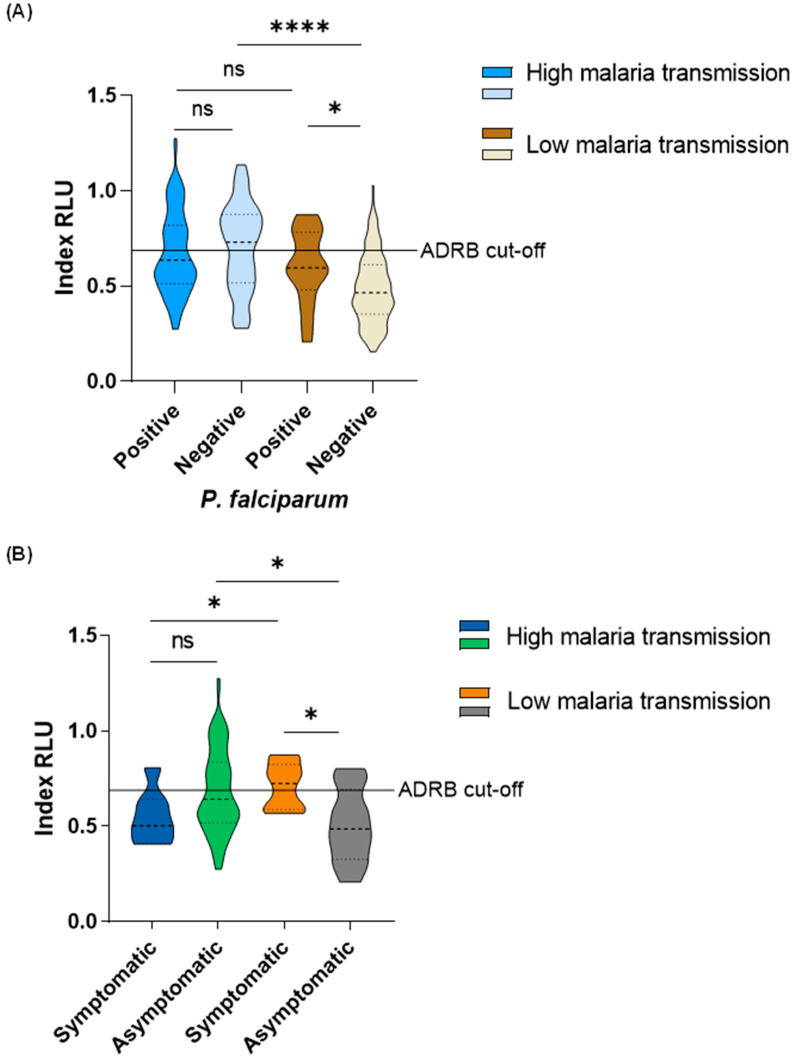
ADRB activity among individuals that were either of the following: (**A**) *P. falciparum*-positive and -negative by PCR in the high and low malaria transmission periods. The number of individuals during the high malaria transmission that are PCR-positive n = 113, PCR-negative n = 45, and during the low malaria transmission that are PCR-positive n = 17 and PCR-negative n = 154. (**B**). *P. falciparum*-positive by PCR with fever (symptomatic) and *P. falciparum*-positive by PCR without fever (asymptomatic) at the time of the survey. The number of individuals during the high malaria transmission that are symptomatic n = 7 and asymptomatic n = 106, and during the low malaria transmission that are symptomatic n = 9 and asymptomatic n = 12. ADRB cut-off is indicated by the solid horizontal line and defined as mean plus three standard deviations indexed RLU of six negative controls. Medians between groups are compared using the Mann–Whitney U test. On the violins, the Median Index RLU is represented by the thick dotted line, while the 25th and the 75th percentiles are represented by the thin dotted lines. The * represents *p* ≤ 0.05, **** represents *p* < 0.0001, and ns represents *p* > 0.05.

During the high malaria transmission period, there was no difference in ADRB activity between *P. falciparum*-positive (median: 0.64 RLU, IQR: 0.51 RLU–0.82 RLU) and -negative (median: 0.73 RLU, IQR: 0.52 RLU–0.88 RLU) individuals (Mann–Whitney U test, *p* = 0.29). Interestingly, during the low malaria transmission period, ADRB activity was higher among *P. falciparum*-positive individuals (median: 0.60 RLU, IQR: 0.52 RLU–0.77 RLU) than in *P. falciparum*-negative individuals (median: 0.47 RLU, IQR 0.36 RLU–0.61 RLU) (Mann–Whitney U test, *p* = 0.01). There was no difference in ADRB activity between *P. falciparum*-positive individuals from the high (median: 0.64 RLU, IQR: 0.51 RLU–0.82 RLU) versus low (median: 0.60 RLU, IQR: 0.52 RLU–0.77 RLU) malaria transmission period (Mann–Whitney U test, *p* = 0.40). However, ADRB activity was higher among *P. falciparum*-negative individuals from the high malaria transmission period (median: 0.73 RLU, IQR: 0.52 RLU–0.88 RLU) than those in the low malaria transmission period (median: 0.47 RLU, IQR 0.36 RLU–0.61 RLU) (Mann–Whitney U test, *p* < 0.0001) (Figure 3A).

There was no difference in ADRB activity between individuals with symptomatic malaria (median: 0.50 RLU, IQR: 0.48 RLU–0.66 RLU) and asymptomatic infections (median: 0.64 RLU, IQR: 0.51 RLU–0.84 RLU) (Mann–Whitney U test, *p* = 0.12) in the high malaria transmission period. However, ADRB activity was higher in individuals with symptomatic malaria (median: 0.72 RLU, IQR: 0.60 RLU–0.80 RLU) than those with asymptomatic infections (median: 0.49 RLU, IQR: 0.37 RLU–0.63 RLU) (Mann–Whitney U test, *p* = 0.018) during the low malaria transmission period. In individuals with symptomatic malaria, higher ADRB activity was observed from those in the low (median: 0.72 RLU, IQR: 0.60 RLU–0.80 RLU) than those in the high (median: 0.50 RLU, IQR: 0.48 RLU–0.66 RLU) (Mann–Whitney U test, *p* = 0.041) malaria transmission period. Nevertheless, in individuals with asymptomatic infection, higher ADRB activity was observed from those in the high (median: 0.64 RLU, IQR: 0.51 RLU–0.84 RLU)) than those in the low (median: 0.49 RLU, IQR: 0.37 RLU–0.63 RLU) (Mann–Whitney U test, *p* = 0.014) malaria transmission period (Figure 3B).

To assess the association between ADRB activity (top versus low ADRB responder) and parasitemia (parasite positivity as measured by PCR), adjusting for age (as a continuous variable), sex, and fever, a logistics regression model stratified by malaria transmission was fitted. The odds were significantly higher among adults than in children in both the high (OR: 1.109, 95% CI: 1.052–1.186, *p* < 0.001) and low (OR: 1.071, 95% CI: 1.038–1.110, *p* < 0.001) malaria transmission period. Also, during the low but not the high malaria transmission period, individuals who were parasite PCR-positive at the time of the survey had significantly higher odds of being ADRB’s top responders (OR: 8.273, 95% CI: 1.851–40.596, *p* = 0.006) than individuals who were parasite PCR-negative at the time of the survey (Table 2).

## 4. Discussion

The development of naturally acquired immunity to *P. falciparum* depends on exposure to the parasite. Consequently, malaria transmission intensity influences the development of naturally acquired immunity to *P. falciparum* [30,31]. In this study, we aimed to evaluate naturally acquired immunity in an area that has experienced a gradual decline in malaria transmission over 25 years from 1993 to 2019. To accomplish this, we assessed the ability of opsonizing antibodies against *P. falciparum* merozoites to recruit neutrophils in an in vitro solid-phase chemiluminescence assay called the antibody-dependent respiratory burst (ADRB). ADRB has a well-standardized protocol [15] and has previously been reported as a correlate of protection against clinical malaria [17]. Here, we measured ADRB activity in children and adults, comparing a period of high versus low malaria transmission.

Indeed, as malaria transmission declined in Nyamisati, ADRB activity toward *P. falciparum* declined. ADRB activity was significantly higher during the high malaria transmission period compared to the low malaria transmission period. Since intensified malaria transmission leads to frequent exposure to *P. falciparum*, this may have promoted the development of long-lived antibody-producing cells and memory-B cells that rapidly produce antibodies following a secondary infection [32]. This, in turn, may have resulted in the recurrent production of antibodies that enhanced ADRB activity. In contrast, the infrequent exposure during the low malaria transmission period limited the boosting of antibodies that may promote ADRB activity. A study in Senegal conforms with our results [16], proposing that antibodies promoting ADRB activity correlate to the degree of malaria endemicity. A different longitudinal study assessing other antibody-mediated functions in children reported a decline in opsonic phagocytosis and maintenance of complement-fixing activity over a year [20]. The decrease in malaria transmission intensity does not necessarily impact all antibody-mediated functions equally; other antibody-mediated functions may be maintained.

In a logistic regression analysis assessing the association between ADRB activity and parasitemia, age predicted ADRB activity. In malaria-endemic areas, an individual’s age reflects cumulative exposure to malaria; consequently, the older one is, the less susceptible they are to severe forms of malaria since naturally acquired immunity to malaria is developed after repeated exposure [18,30]. This suggests that acquiring antibodies promoting ADRB activity coincides with developing naturally acquired immunity rendered by exposure. Other studies have reported this observation assessing ADRB activity [16,17] and other Fc-dependent antibody-mediated functions against *P. falciparum* [33,34]. A captivating observation in our study was that only adults 26 years and above during the high malaria transmission period had median ADRB activity above the ADRB cut-off. This observation may imply that as malaria transmission declined in Nyamisati, naturally acquired immunity against *P. falciparum* may have declined. Declines in the level of immunity in a population may influence the occurrence of malaria epidemics with a significant morbidity burden. One study in Thailand implied that the decrease in *P. falciparum* transmission and the level of immunity might influence the emergence of artemisinin-resistant falciparum malaria [19]. Parts of Africa have experienced a significant reduction in malaria transmission [35], and if the levels of immunity decline, malaria vaccines promoting strong antibody-mediated functions against *P. falciparum* will be necessary to maintain immunity.

We observed higher ADRB activity among *P. falciparum*-positive than -negative individuals during the low but not the high malaria transmission period. Median ADRB activity among *P. falciparum*-positive individuals during the low malaria transmission period was similar to median ADRB activity observed during the high malaria transmission period. This observation could be because ongoing *P. falciparum* infection boosted antibodies promoting ADRB activity [36,37] to similar levels observed during the high malaria transmission period. The boosting of antibodies that may promote antibody-mediated functional activity has been reported with antibody-mediated opsonic phagocytosis [26] and antibody-dependent natural killer cell activity [38] against *P. falciparum* in Kenyan children. Antibody boosting is not likely to significantly influence differences in ADRB activity among parasite-positive versus -negative individuals in the high malaria transmission period due to constant exposure to the parasite.

The finding that symptomatic malaria during the survey was a significant predictor of higher ADRB activity during the low malaria transmission period cannot be explained. This finding contradicts longitudinal studies reporting ADRB activity against *P. falciparum* as a correlate of protection against clinical malaria [16,17]. Further studies are necessary to assess the correlation between antibody-mediated functions and the risk of malaria in this cohort.

Our study has some limitations. Firstly, the analyses are based on data from cross-sectional surveys; therefore, inferences about protection against malaria on an individual level cannot be made. In addition, samples were collected over a long period, which could have influenced the quality of archived samples. Nonetheless, immunoglobulins are known to be stable, and their integrity is not significantly affected by long-term storage [39]. Lastly, we employed only ADRB to assess the functional aspects of antibody-mediated immunity to *P. falciparum*. Since there is no single correlate measure of immunity to malaria, other antibody-mediated functions could be used with ADRB to assess the level of immunity to malaria. Besides antibody-mediated functional activity, antibody subclass, specifically the cytophilic IgG1 and IgG3 [40,41], and antibody levels to specific *P. falciparum* antigens [42] have been predictors of immunity against clinical malaria. Assessing these with antibody-dependent functional activity will provide a finer understanding of immunity to malaria.

In summary, we show that in Nyamisati, an area with declining malaria transmission in Tanzania, antibodies promoting ADRB activity against *P. falciparum* reduce in both children and adults and are influenced by exposure to the parasite. These findings propose that naturally acquired immunity to *P. falciparum* decreased in children and adults as malaria transmission declined. The implications, therefore, are that the village may be susceptible to higher malaria morbidity in case of a surge in transmission, emphasizing the need for effective malaria vaccines. With the increased efforts to control malaria, it is necessary to prioritize studies assessing the impact of decreased malaria transmission on naturally acquired immunity. These studies will help underline the necessity of effective malaria vaccines in preventing the rebound of malaria, thereby aiding in eliminating malaria.

## Figures and Tables

**Table 1:** Characteristics of study population participating in high versus low malaria transmission periods.

		High Malaria Transmission Period	Low Malaria Transmission Period
Survey years		1993, 1994, 1995	2016, 2018, 2019
Individuals, n		158	171
Age, years	Median (IQR)	16 (10–31)	16 (9–29)
Age groups, n	0–4	10	13
	5–8	25	27
	9–12	23	28
	13–16	22	23
	17–20	10	16
	21–25	19	14
	26–30	9	11
	31–35	11	9
	36–40	13	12
	>40	16	18
Sex, n (% [95% CI])	Female	88 (55.7 [47.9–63.2])	104 (60.8 [53.3–67.8])
	Male	70 (44.3 [0.37–0.52])	67 (39.2 [0.32–0.47])
Fever at sampling, n (% [95% CI])		8 (5.1 [2.6–9.7])	41 (24 [18.2–30.9])
Microscopy positive, n (% [95% CI])	All	60 (37.9 [30.8–45.7])	9 (5.3 [2.8–9.7])
	Asymptomatic	52 (32.9 [26.2–40.6])	4 (2.3 [0.9–5.9])
	Symptomatic	8 (5.1 [2.6–9.7])	5 (2.9 [1.3–6.7])
*P. falciparum* parasite densities, p/µL (IQR) by microscopy	All	360 (80–800)	760 (560–3160)
	Asymptomatic	280 (80–560)	1520 *
	Symptomatic	2760 (1940–7220)	3160 (560–15,360)
Real-time PCR positive, n (% [95% CI])	All	120 (75.9 [68.7–81.9])	21 (12.3 [81.7–18.0])
Real-time PCR positive *P. falciparum,* n (% [95% CI])	All	113 (71.5 [64–78])	17 (9.9 [6.3–15.3])
	Asymptomatic	106 (67.1 [59.4–73.9])	8 (4.7 [2.4–9])
	Symptomatic	7(4.4 [2.2–8.9])	9(5.3 [2.8–9.7])

* There was only one individual with an asymptomatic infection and detectable parasite density by microscopy in the low malaria transmission period.

**Table 2:** Association between ADRB and parasite-positive (as measured by PCR), adjusted for age, sex, and fever.

	Variables	OR	95% CI	*p* Value
High malaria transmission period	Age	1.109	1.052–1.186	**<0.001**
	Sex, Female	0.927	0.339–2.492	0.957
	Parasite-positive	1.033	0.315–3.219	0.956
	Febrile	1.059	0.112–10.073	0.957
Low malaria transmission period	Age	1.071	1.038–1.110	**<0.001**
	Sex, Female	0.694	0.258–1.858	0.464
	Parasite-positive	8.273	1.851–40.596	**0.006**
	Febrile	0.676	0.191–2.112	0.518

The statistically significant *p* values are written in bold.

## Data Availability

The data presented in this study are available upon request from the corresponding author. The data are not publicly available due to ethical restrictions.

## Supplementary Materials

The following supporting information can be downloaded at https://www.mdpi.com/article/10.3390/vaccines12020203/s1, Figure S1: Median age among individuals that are *P. falciparum*-positive by PCR with fever (symptomatic) and *P. falciparum*-positive by PCR without fever (asymptomatic) at the time of the survey during the high and low malaria transmission period. On the violins, the Median age is represented by the thick dotted line, while the 25th and the 75th percentiles are represented by the thin dotted lines. The * represents *p* ≤ 0.05, **** represents *p* < 0.0001, and ns represents *p* > 0.05. The number (n) of individuals in each symptomatic and asymptomatic group in the high and low malaria transmission period is presented in Table 1. Table S1: Distribution of study participants by their ADRB status in age categories.
