# Supplementary material for: Antibody-Dependent Respiratory Burst against Plasmodium falciparum Merozoites in Individuals Living in an Area with Declining Malaria Transmission
Source: Vaccines (Basel). 2024 Feb 16;12(2):203. doi: 10.3390/vaccines12020203 (PMC10892224; doi:10.3390/vaccines12020203)
Supplement: Supplementary file 1 [file vaccines-12-00203-s001.zip › Mutemi_et_al_Table_S1.pdf]

Table S1. Distribution of study participants by their ADRB status in age categories

| High malaria transmission | | | | Low malaria transmission | | |
| --- | --- | --- | --- | --- | --- | --- |
| Age groups (years) | ADRB positive (n) | ADRB negative (n) | **Total** | ADRB positive (n) | ADRB negative (n) | **Total** |
| 0-4 | 2 | 8 | **10** | 1 | 12 | **13** |
| 5-8 | 4 | 21 | **25** | 0 | 27 | **27** |
| 9-12 | 11 | 12 | **23** | 5 | 23 | **28** |
| 13-16 | 11 | 11 | **22** | 1 | 22 | **23** |
| 17-20 | 4 | 6 | **10** | 3 | 13 | **16** |
| 21-25 | 9 | 10 | **19** | 4 | 10 | **14** |
| 26-30 | 7 | 2 | **9** | 3 | 8 | **11** |
| 31-35 | 6 | 5 | **11** | 2 | 7 | **9** |
| 36-40 | 7 | 6 | **13** | 4 | 8 | **12** |
| >40 | 11 | 5 | **16** | 6 | 12 | **18** |
| **Total** | **72** | **86** | **158** | **29** | **142** | **171** |
