# Supplementary figures and images for: Antibody-Dependent Respiratory Burst against Plasmodium falciparum Merozoites in Individuals Living in an Area with Declining Malaria Transmission
Source: Vaccines (Basel). 2024 Feb 16;12(2):203. doi: 10.3390/vaccines12020203 (PMC10892224; doi:10.3390/vaccines12020203)

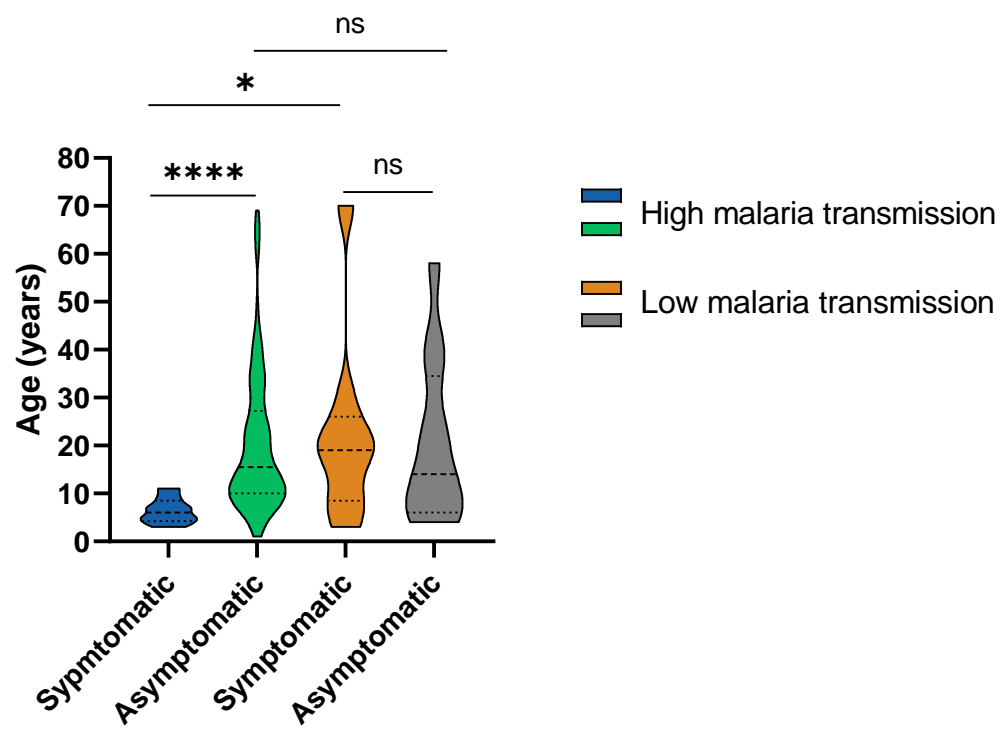

Supplement: Supplementary file 1 [file vaccines-12-00203-s001.zip › Mutemi_et_al_SuppFigureS1.pdf]
